# Factors associated with eating behaviors of young and middle-aged adults with overweight and obesity: a systematic review

**DOI:** 10.3389/fpubh.2026.1785677

**Published:** 2026-05-05

**Authors:** Yan He, Yanjie Liu, Wanya Pan, Wenhao Tian, Yuan Zhao, Yunyu Guo, Xiuqin Feng

**Affiliations:** Department of Nursing, The Second Affiliated Hospital, Zhejiang University School of Medicine, Hangzhou, China

**Keywords:** eating behavior, middle-aged adults, obesity, overweight, systematic review, young adults

## Abstract

The escalating prevalence of overweight and obesity among young and middle-aged adults constitutes a significant public health challenge. While modifying dietary behavior is accepted as a critical component of weight management, existing reports regarding the specific drivers of these behaviors remain scattered and lack systematic integration. To address this knowledge gap, this review systematically synthesized the factors associated with eating behaviors within this demographic. Adhering strictly to the Preferred Reporting Items for Systematic Reviews and Meta-Analyses protocol, a comprehensive search across seven electronic databases was conducted, identifying nine eligible cross-sectional studies comprising 7,614 participants. Narrative synthesis categorized the identified associated factors into three overarching themes: psycho-emotional factors, encompassing negative affective states, impulsivity, and emotion regulation deficits; socio-environmental stressors defined by occupational environments and interpersonal dynamics; and maladaptive behavioral patterns such as rapid eating rates and irregular meal timing. These findings critically indicate that eating behaviors in this population often serve as compensatory mechanisms for emotional and environmental strain rather than solely reflecting nutritional choices. Consequently, effective weight management strategies must extend beyond traditional dietary education to incorporate holistic approaches involving emotion regulation, stress management, and behavioral modification. By addressing these upstream non-nutritional determinants and psychological distress, healthcare providers can develop more comprehensive, targeted, and successful interventions to support weight management efforts in young and middle-aged adults.

## 1 Introduction

Overweight and obesity have emerged as a critical global public health challenge, with prevalence rates rising steadily over recent decades, particularly among young and middle-aged adults (1, 2). Prioritizing this demographic is essential, as managing weight trajectories during these high-stress peak working years can help mitigate the negative impact of cumulative stress and prevent long-term cardiometabolic consequences (3). According to estimates from the World Health Organization, nearly 60% of the adult population worldwide is now affected by excess adiposity (4), reflecting a widespread shift toward unhealthy weight trajectories across diverse socioeconomic and cultural contexts. This growing burden is a well-established contributor to major noncommunicable diseases, including type 2 diabetes and cardiovascular disease (5, 6). placing sustained pressure on healthcare systems and generating significant economic costs through productivity loss and long-term disability (7).

Eating behavior plays a central role in both the development and management of obesity, representing a modifiable determinant of nutritional status (8). In this review, eating behavior is defined as the psychological and behavioral patterns surrounding food intake, encompassing dimensions such as emotional eating, external eating, and dietary restrain (9). Young and middle-aged adults, often described as the “sandwich generation” due to the dual pressures of career development and caregiving for both children and aging parents (10), face unique challenges. The cumulative burden of these competing demands is closely linked to psychological stress and time pressure, which are associated with disruptions in healthy eating patterns. In such contexts, homeostatic hunger cues may be overridden, and eating often emerges as a coping response to external pressures rather than physiological need (11, 12). Observational studies utilizing validated tools, such as the Dutch Eating Behavior Questionnaire, have indicated that the prevalence of emotional eating among adults with overweight and obesity in community and clinical settings typically ranges between 40% and 60% (13). Consequently, sustainable weight management may require shifting focus from caloric restriction alone toward the complex determinants driving these maladaptive behaviors.

Despite its importance, the existing evidence base remains fragmented (14, 15). Previous systematic reviews have focused predominantly on pediatric and adolescent populations, emphasizing parental feeding practices (16, 17). However, these findings offer limited insight into adult populations, who exercise greater autonomy and face distinct occupational stressors (18). Furthermore, while existing adult-focused studies have classified eating styles or linked them to body mass index, fewer studies have systematically integrated the “upstream” predictors of these behaviors, such as personality traits and environmental cues (19, 20). To address this, our review employs a biopsychosocial framework to systematically organize these multifaceted “upstream” factors into interactive psychological, socio-environmental, and behavioral categories. The absence of such a synthesis limits the development of individualized weight-management interventions. Therefore, this systematic review aims to address this gap by providing a novel synthesis of the multifaceted factors associated with eating behaviors specifically among young and middle-aged adults (defined as individuals aged 18–60 years) with overweight and obesity. By comprehensively mapping these underlying correlates, we aim to inform more targeted, evidence-informed approaches to care.

## 2 Methods

In this study, we employed a systematic review approach to evaluate and synthesize existing literature, ensuring rigor and reliability in the results. This review followed the guidelines of the Cochrane Effective Practice and Organization of Care Group (8) and adhered to the Preferred Reporting Items for Systematic Reviews and Meta-Analyses (PRISMA) statement (21). The PRISMA checklist was used, covering key sections such as eligibility criteria, information sources, search strategy and study selection. The protocol for this review was registered on PROSPERO (CRD420251229603), And can be accessed at https://www.crd.york.ac.uk/PROSPERO/view/CRD420251229603.

### 2.1 Data sources

A systematic search was conducted in seven electronic databases: PubMed, Web of Science, Embase, CINAHL, Cochrane, China National Knowledge Infrastructure, and WanFang Database from inception to 17 November 2025. These databases were selected to provide comprehensive coverage of both international and regional literature, thereby reducing language and publication biases and ensuring a global perspective on the target population (22, 23).

### 2.2 Search strategy

The search strategy was developed using Medical Subject Headings (MeSH) and free-text terms. The core syntax utilized Boolean operators (AND/OR) and was adapted for each database. Keywords included: (“young adult” OR “middle-aged” OR “youth” OR “young people” OR “young adults”) AND (“obesity” OR “obese” OR “fat” OR “overweight” OR “adipositas” OR “adiposity” OR “obesitas”) AND (“feeding behavior” OR “eating behavior^*^” OR “diet behavior^*^” OR “dietary behavior^*^” OR “food behavior^*^”) AND (“risk factors” OR “risk Score^*^” OR “risk factor score^*^” OR “social risk factor^*^” OR “health correlates” OR “influencing factor^*^” OR “related factor^*^”). Broad search terms such as ‘health correlates' and ‘risk factors' were intentionally utilized to ensure a comprehensive, exploratory search that captured unanticipated ‘upstream' variables across the biopsychosocial spectrum. No date or language restrictions were applied during the search stage to ensure maximum retrieval. Detailed search strategies tailored to each database are provided in Appendix 1.

### 2.3 Selection criteria

#### 2.3.1 Eligibility criteria

Studies were included if they met the following criteria:(1) participants aged 18–60 years, defined in this review as young adults aged 18–35 years and middle-aged adults aged 36–60 years; (2) studies must have included participants with overweight or obesity (defined according to World Health Organization criteria or region-specific standards) and provided separate data analyses for this subgroup, for studies with mixed BMI samples (general population), only data specific to the overweight/obesity subgroup were extracted; (3) studies reporting on factors associated with eating behaviors, where eating behavior was assessed using validated scales or questionnaires [i.e., psychometrically established instruments (24), such as the DEBQ or TFEQ]; (4) observational studies, including cross-sectional, cohort, and case-control designs; and (5) studies published in Chinese or English.

#### 2.3.2 Exclusion criteria

Studies were excluded if they involved: (1) pregnant or lactating women, as well as participants diagnosed with severe organic diseases such as severe gastrointestinal conditions, or metabolic disorders including untreated hypothyroidism and Cushing's syndrome; (2) duplicate publications or studies where the full text was unavailable; (3) reviews (including systematic reviews and meta analyses), animal studies, comments, and conference or case reports.

### 2.4 Study selection

**Figure 1:**
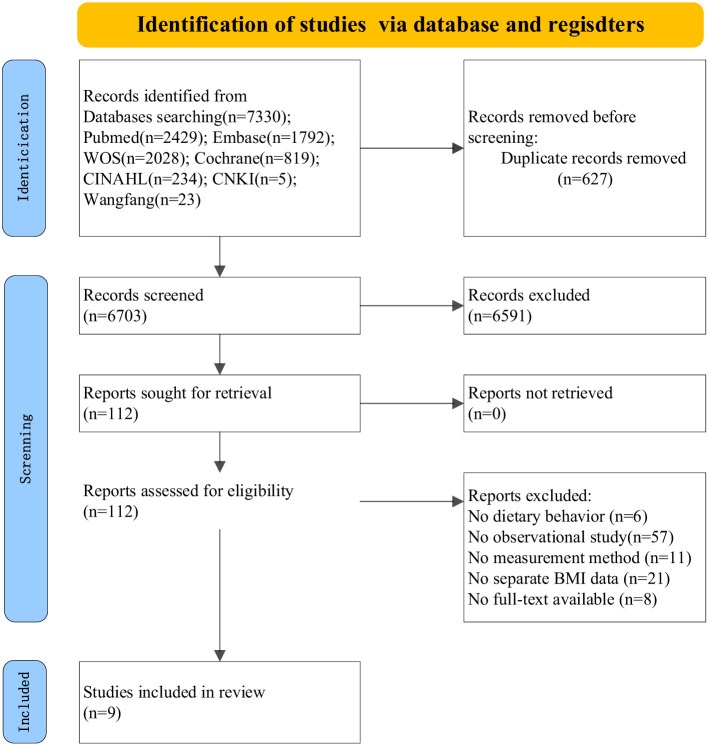
Flowchart of article inclusion in the systematic review.

Utilizing Zotero 7.0 software, two researchers independently performed the literature screening. First, titles and abstracts were screened to exclude irrelevant studies. Second, full texts of potentially eligible articles were retrieved and assessed against the inclusion criteria. Discrepancies at either stage were resolved through discussion; if a consensus could not be reached, a third researcher was consulted. Figure 1 illustrates the step-by-step search and selection process.

### 2.5 Quality assessment

We used the 11-item questionnaire recommended by the Agency for Healthcare Research and Quality (AHRQ) (25) to evaluate methodological quality, as it is specifically designed for the risk-of-bias assessment of cross-sectional observational studies. Two researchers independently assessed each study, with cross-checking for consistency. Disagreements were resolved through discussion with a third reviewer. For each item, a response of “Yes” received 1 point, while “No” or “Unclear” received 0 points, resulting in a total possible score of 11. The quality of the articles was classified as follows: scores ≤ 3 indicated low quality, 4–7 indicated moderate quality, and ≥ 8 indicated high quality (26). Quality scores were used to contextualize the strength of the evidence during the synthesis but were not used as a basis for exclusion. Further details are provided in Table 1.

**Table 1:** Quality assessment using the AHRQ checklist.

Studies included	①	②	③	④	⑤	⑥	⑦	⑧	⑨	⑩	⑪	Score
Elfhag (28)	1	0	1	0	1	0	0	1	0	0	0	4
Ozier (12)	1	0	1	0	0	0	1	1	0	0	0	4
Nishitani (11)	1	1	1	1	0	0	1	0	1	0	0	6
Sonneville (32)	1	1	1	1	1	0	1	1	0	0	0	7
Fox (30)	1	0	1	0	0	0	1	0	1	0	0	4
Braden (29)	1	1	0	0	1	1	1	1	0	0	0	6
Canterini (33)	1	1	1	1	1	0	1	1	1	0	0	8
Yan (31)	1	1	0	0	1	1	1	1	0	0	0	6
Jacob (34)	1	1	1	1	1	1	1	1	1	1	0	10

①Define the source of information (survey, record review). ②List inclusion and exclusion criteria for exposed and unexposed subjects (cases and controls) or refer to previous publications. ③Indicate time period used for identifying patients. ④Indicate whether or not subjects were consecutive if not population-based. ⑤Indicate if evaluators of subjective components of study were masked to other aspects of the participants. ⑥Describe any assessments undertaken for quality assurance purposes (e.g., test/retest of primary outcome measurements). ⑦Explain any patient exclusions from analysis. ⑧Describe how confounding was assessed and/or controlled. ⑨If applicable, explain how missing data were handled in the analysis. ⑩Summarize patient response rates and completeness of data collection. ⑪Clarify what follow-up, if any, was expected and the percentage of patients for which incomplete data or follow-up was obtained.

### 2.6 Data extraction

Two researchers independently extracted key information from the included articles using a standardized data extraction form. Extracted information included: study characteristics (author, year, country, design), participant demographics (sample size, age, sex, BMI categories), eating behavior outcomes and main findings. Notably, absolute mean behavioral scores were not extracted due to the substantial heterogeneity in scoring systems across different questionnaires, which precluded meaningful direct comparison. Discrepancies were resolved through discussion or consultation with a third researcher. Further details are provided in Table 2.

**Table 2:** Characteristics and synthesized findings of the included studies.

Reference	Country	Mean age	Female (%)	Mean BMI	BMI cut-off values for overweight and obesity	Sample size	Sampling method	Eating behavior assessment tool	Main findings
Elfhag (28)	Sweden	43.7 years (SD 12.6)	63.8%	Mean 40.5 kg/m^2^ (SD 5.3)	Obesity defined as BMI ≥30 kg/m^2^	442	Convenience sampling	Dutch eating behavior; questionnaire (DEBQ)	Emotional eating correlated most strongly with Neuroticism-Impulsiveness (*r* = 0.49) and negatively with Conscientiousness-Self-discipline (*r* = −0.33); the same personality pattern, albeit weaker, was seen for external eating, whereas restrained eating showed the reverse profile.
Ozier (12)	USA	45.2 years (SD 11.6)	73.5%	Mean 27.3 kg/m^2^ (SD 6.4)	Overweight/obesity defined as BMI ≥25 kg/m^2^	822	Convenience sampling	Revised NEO personality inventory (NEO PI-R)	In the final logistic model (R^2^ = 0.265) the lowest-scoring quartile of Factor-1 “Emotion and Stress-Related Eating” had 13.4–fold higher odds of being overweight/obese compared with the highest-scoring quartile (95 % CI 8.0–22.3); intermediate quartiles showed dose-response ORs of 3.1 and 2.0. Race, sex, job category and life-stage were retained covariates.
Nishitani (11)	Japan	41.8 years (SD 12.6)	100%	obesity sample Mean 27.5 kg/m^2^ (SD 2.1)^a^	Overweight/obesity defined as BMI ≥25 kg/m^2^	164 obese	Workplace-based census survey	Eating and appraisal due to emotions and stress questionnaire (EADES)	Obesity was cross–sectionally associated with higher scores on “eating to satiety”, “eating fast”, “substitution eating when irritated”, and “feeling of satiety” (age-adjusted OR 1.16–1.93; all p ≤ 0.001). Quantitative workload correlated most strongly with these eating patterns (ρ = 0.25–0.35), while psychological stress responses (fatigue, tension/anxiety, depression) showed slightly weaker but consistent positive correlations (ρ = 0.20–0.40).
Sonneville (32)	USA	21.4–21.7 years (SD 0.12–0.31)	49%	Mean 27.7 kg/m^2^ (SD 0.17–0.60)	Overweight/obesity defined as BMI ≥25 kg/m^2^	5184	Stratified, multi-stage probability sample	Sakata eating behavior questionnaire(SEBQ)	Weight under-perception was associated with markedly lower prevalence of fasting/skipping meals (F 17 % vs 19.5 %; M 4.2 % vs 12.9 %), vomiting/diet-pill use (F 1 % vs 9.4 %; M 0.6 % vs 2 %) and, in females only,
									overeating/loss-of-control episodes (4.8 % vs 11 %). Conversely, under-perceivers of both sexes showed ~ 2-fold higher use of performance-enhancing substances. All estimates were adjusted for age, BMI, race/ethnicity, poverty ratio and parental education.
Fox (30)	Ireland	47.0 years (SD 14.7)	70.3%	Mean 31.2 kg/m^2^ (SD 5.6)	Overweight/obesity defined as BMI ≥25 kg/m^2^	97	Convenience sampling	Job stress questionnaire	Negative attitudes toward emotional expression predicted higher emotional-eating scores.
Braden (29)	USA	41.78 years (SD 13.61)	64.9%	Mean 33.17 kg/m^2^ (SD 6.98)	Overweight/obesity defined as BMI ≥25 kg/m^2^	188	Convenience sampling	Self-reported single-item weight-perception question	(β = 0.59) and, together with the diffuse-emotion eating pattern, explained 11% of variance in BMI (β ≈ 0.37–0.39).
Canterini (33)	France	38.4 years (SD 12.7)	100%	Mean 45.5 kg/m^2^ (SD 6.7)	Obesity defined as BMI ≥35 kg/m^2^	116	Convenience sampling	Four brief Add Health eating-behavior items	EE-Depression subscale uniquely predicted poorer psychological wellbeing (β = 0.40), eating-disorder symptoms (β = 0.36) and emotion-regulation difficulties (β = 0.32); EE-positive emotions was unrelated to any outcome.
Yan (31)	China	20.37 years (SD 1.31)	71.7%	Mean 29.82 kg/m^2^ (SD 4.63)	Overweight/obesity defined as BMI ≥24 kg/m^2^	300	Convenience sampling	Binge eating scale (BES)	“Rapid eating” (score ≥ 7/10) was present in 50% and correlated with higher emotional-eating and external-eating scores (*r* = 0.30 each); rapid eaters more often reported “eating too much” (63% vs 25%, *p* < 0.001).
Jacob (34)	Canada	38.7 years years (SD 8.5)	55.8%	Mean 33.2 kg/m^2^ (SD 3.4)	Overweight/obesity defined as BMI ≥25 kg/m^2^	301	Convenience sampling	Dutch eating behavior questionnaire (DEBQ)	Seventeen percent scored ≥ 17 (moderate–severe binge eating). Female sex (OR 1.05), previous uncontrolled eating (OR 6.04), weight-loss dieting (OR 2.64), peer competition (OR 2.59) and interpersonal distress (OR 2.12) were independently associated with higher binge-eating severity; BMI, academic stress, body-shape teasing and life events were correlated in univariate analyses only.

BMI≥25.0 kg/m^2^ per Japanese (JASSO) criteria, this sample was categorized within the overweight/obesity subgroup.

### 2.7 Data synthesis

A meta-analysis was not conducted due to substantial methodological and clinical heterogeneity, stemming from the use of diverse assessment instruments (e.g., DEBQ, TFEQ-R18), varying definitions of eating behavior dimensions, and differences in participant characteristics; instead, a conventional content analysis approach was adopted (27). In terms of epistemological positioning, the researchers adopted a subtle realist approach. This perspective acknowledges that while eating behaviors and their associated correlates exist as measurable phenomena, the overarching themes must be generated through the researchers' interpretive synthesis. To clarify the process of thematic development and analysis, a systematic three-step coding procedure was utilized: (1) line-by-line extraction of statistically significant behavioral determinants; (2) inductive clustering of these variables into descriptive subthemes; and (3) generation of overarching analytical themes. This framework was developed inductively by two researchers who independently reviewed the extracted data, and consensus on the final thematic structure was achieved through iterative cross-checking and discussion. We acknowledge that treating unavailable quantitative data as ‘qualitatively reported' may introduce bias, though this approach was necessary to ensure a comprehensive synthesis of the available evidence. Further details are provided in Table 3.

**Table 3:** Factors associated with eating behaviors in young and middle-aged adults with overweight and obesity.

Theme	Subtheme	Number of studies	Studies included
Psycho-emotional factors	Negative affective states	4	Eifhag et al. (28); Ozier et al. (12); Nishitani et al. (11); Braden et al. (29); Jacob et al. (34)
	Personality traits and impulsivity	2	Eifhag et al. (28); Fox et al. (30);
	Emotion regulation deficits	3	Ozier et al. (12); Jacob et al. (34); Braden et al. (29)
Socio-environmental stressors	Occupational environment	2	Nishitani et al. (11); Jacob et al. (34)
	Interpersonal dynamics	1	Yan et al. (31)
	Broader sociodemographic context	1	Sonneville et al. (32); Ozier et al. (12)
Maladaptive behavioral and cognitive patterns	Eating rate and satiety responsiveness	2	Canterini et al. (33); Nishitani et al. (11)
	Meal timing and circadian rhythm	1	Jacob et al. (34)
	Weight–perception and dieting history	2	Sonneville et al. (32); Yan et al. (31)

“Number of Studies”refers to the frequency of included articles that reported a significant association with the respective subtheme.

## 3 Results

### 3.1 Search results

A total of 7,614 young and middle-aged adults with overweight and obesity were represented across the nine quantitative studies included in this systematic review. Geographically, the research spanned diverse cultural contexts, including the United States (*n* = 3), Canada (*n* = 1), China (*n* = 1), Japan (*n* = 1), Ireland (*n* = 1), France (*n* = 1), and Sweden (*n* = 1). Due to the limited number of studies per region, geographical context was not utilized for comparative interpretation; rather, the synthesis focused on identifying cross-cutting behavioral patterns across these demographic settings. The methodological quality, assessed via the AHRQ scale, was moderate overall, with scores ranging from 4 to 10. Two studies achieved a high-quality rating (≥8 points), while seven were classified as moderate (4–7 points). The primary methodological caveats identified included inadequate descriptions of missing data handling, lack of blinding for outcome assessors, and insufficient adjustment for potential confounders, which should be considered when interpreting the strength of the evidence. Given these variations in study design and the substantial heterogeneity in behavioral assessment tools, no quantitative pooling was performed; findings are therefore presented via narrative synthesis.

### 3.2 Synthesis of themes from the literature

Eating behavior in this population is shaped by a variety of factors, which are categorized into three overarching themes and nine specific subthemes. Theme 1 includes psycho-emotional factors, such as encompassing negative affective states, personality traits and impulsivity perceived stress and emotion regulation deficits; Theme 2 encompasses socio-environmental stressors, including characterized by occupational environment, interpersonal dynamics, broader sociodemographic context Theme 3 consists of maladaptive behavioral and cognitive patterns, such as eating rate and satiety responsiveness, meal timing and rhythm, weight perception and dieting history. Details provided in Table 3.

#### 3.2.1 Psycho-emotional factors

##### 3.2.1.1 Negative affective states

Negative affective states, encompassing stress, depression, anxiety, and boredom, were consistently associated with dysregulated eating behaviors, particularly emotional eating and loss of control. The evidence suggests a graded association, for instance, Elfhag et al. (28) reported that individuals in the highest quartile for “Emotion and Stress-Related Eating” had significantly higher odds of obesity compared to those in the lowest quartile (OR = 13.4). Similarly, Braden et al. (29) found that depression-driven emotional eating was associated with both diminished psychological wellbeing and increased severity of eating disorder symptoms. These findings highlight that negative affect functions as a significant correlate of eating pathology in this demographic.

##### 3.2.1.2 Personality traits and impulsivity

Beyond transient mood states, certain personality traits were linked to an individual's responsiveness to external and emotional cues. A coherent profile emerged across several studies: higher neuroticism and impulsivity were positively correlated with emotional eating, whereas conscientiousness showed an inverse relationship. Elfhag et al. (28) demonstrated a strong positive correlation between emotional eating and a “Neuroticism–Impulsiveness” factor (*r* = 0.49), and an inverse relationship with Conscientiousness–Self-discipline (*r* = −0.33). These associations suggest that maladaptive eating may be linked to broader difficulties in self-regulation within emotionally salient contexts.

##### 3.2.1.3 Emotion regulation deficits

The relationship between psychological distress and overeating appears to be linked to deficits in emotion regulation. Maladaptive regulatory strategies, restricted emotional expression, and alexithymia were consistently associated with higher BMI and emotional eating scores. Negative attitudes toward emotional expression were found to correlate with higher emotional eating, explaining significant variance in BMI when combined with diffuse emotional patterns (30). Furthermore, Braden et al. (29) observed that difficulties in emotion regulation were associated with depression-driven eating. Collectively, these results suggest that when emotional experiences are difficult to identify or manage, individuals may be more likely to utilize food as a coping strategy.

#### 3.2.2 Socio-environmental stressors

##### 3.2.2.1 Occupational environment

Work-related demands emerged as a salient contextual factor, with evidence linking occupational strain to specific eating patterns. Nishitani et al. (11) observed that workplace environments characterized by high quantitative workload and time pressure were associated with behavioral dysregulation. In their analysis, obesity was significantly associated with “eating fast” and substitution eating when irritated (*p* ≤ 0.001), with quantitative workload showing the strongest correlations with these behaviors. This suggests that high job demands may coincide with structural barriers to healthy eating, where individuals might prioritize speed or immediate stress relief over satiety monitoring.

##### 3.2.2.2 Interpersonal dynamics

Interpersonal stressors, particularly those involving social evaluation and conflict, were linked to more severe eating pathology in the included studies. The findings suggest that relational contexts may heighten affective load, which is associated with dysregulation. In the study by Yan et al. (31), both peer competition (OR = 2.59) and interpersonal distress (OR = 2.12) independently predicted greater binge-eating severity. This suggests a potential link where interpersonal friction may undermine self-regulation, with food serving as an accessible response to manage distress arising from social threats or perceived inferiority.

##### 3.2.2.3 Broader sociodemographic context

Sociodemographic characteristics appeared to modify the relationship between stress and eating behavior. Sonneville et al. (32) highlighted the importance of structural context, showing that associations between weight perception and unhealthy weight-control behaviors remained robust after adjusting for race/ethnicity, poverty ratio, and parental education. This indicates that eating outcomes are situated within resource accessibility and cultural norms. Furthermore, biological sex was identified as a moderator in specific contexts; for example, Yan et al. (31) found female sex to be independently associated with higher binge-eating severity (OR = 1.05). Synthesized across studies, these findings suggest that sociodemographic factors may influence baseline exposure to stress and the availability of coping resources.

#### 3.2.3 Maladaptive behavioral and cognitive patterns

##### 3.2.3.1 Eating rate and satiety responsiveness

A consistent behavioral pattern identified across studies was a perceived mismatch between intake and satiety, often manifested as rapid eating. Canterini et al. (33) found that rapid eating (≥7/10 on a subjective scale) was highly prevalent (50%) and significantly correlated with both emotional and external eating (*r* = 0.30). Crucially, rapid eaters were more than twice as likely to report eating too much compared to their slower counterparts (63% vs. 25%, *p* < 0.001). Nishitani et al. (11) similarly linked obesity to higher scores for eating to satiety and speed. These results indicate that accelerated eating rates are a common marker of dysregulation in emotionally or environmentally demanding contexts.

##### 3.2.3.2 Meal timing and rhythm

Disruptions in meal timing and irregular rhythms were identified as behavioral markers associated with higher energy intake. Jacob et al. (34) provided evidence that meal timing is linked to eating traits, showing that energy consumed after 17:00 was associated with increased total daily caloric intake. Furthermore, late-night intake (after 20:00) correlated with higher scores for disinhibition and susceptibility to hunger. These findings suggest that delayed eating rhythms may reflect a broader pattern of behavioral disinhibition, potentially linked to the accumulation of daily stress or fatigue.

##### 3.2.3.3 Weight perception and dieting history

Cognitive factors, specifically body appraisal and weight control history, were associated with current eating behaviors. Sonneville et al. (32) observed that weight under-perception (perceiving oneself as lighter than actual weight) was associated with a lower prevalence of extreme measures like vomiting but corresponded with other risks, such as overeating episodes. Conversely, a history of restrictive attempts was linked to subsequent dysregulation: Yan et al. (31) reported that a history of uncontrolled eating (OR = 6.04) and previous weight-loss dieting (OR = 2.64) were the strongest predictors of current binge-eating severity. This suggests an association between prior restrictive behaviors and a heightened risk of loss of control.

## 4 Discussion

This systematic review synthesizes the multifaceted determinants associated with eating behavior in young and middle-aged adults with overweight and obesity. Our findings suggest that dysregulated eating is not merely a failure of nutritional knowledge but is associated with a complex biopsychosocial framework where eating behavior may emerge from the interplay of upstream psycho-emotional factors, pervasive socio-environmental stressors, and entrenched maladaptive patterns. The synthesis suggests a potential pathway where eating behavior may function not merely for energy homeostasis, but as a hypothesized compensatory mechanism to modulate affective distress and manage challenges in inhibitory control. Specifically, our review confirms a graded relationship between negative affective states and maladaptive eating, which is consistent with the Affect Regulation Theory of binge eating (35, 36). However, a critical nuance emerges: vulnerability appears linked less to the presence of negative emotions per se than to deficits in emotion regulation—specifically alexithymia and maladaptive coping styles (29, 30). The evidence suggests that for individuals with limited adaptive regulatory skills, food may serve as a “prosthetic” tool to potentially manage aversive states or internal distress. This distinction is vital for clinical interpretation, suggesting that the association between stress and overeating may be linked to a regulatory gap (37). When emotional awareness is low, the physiological sensation of anxiety might be misidentified as hunger, potentially leading to inappropriate caloric intake. Consequently, traditional dietary education may have limited efficacy for this subgroup if it does not address underlying regulatory challenges. Clinical considerations could include the integration of skill-based emotion regulation, such as elements of Dialectical Behavior Therapy (DBT) (38, 39), opening new therapeutic pathways that prioritize emotional skill-building as a prerequisite for successful weight management. Closely intertwined with these emotional vulnerabilities are distinct personality traits. Building upon these transient emotional states, the review also identifies a potential risk phenotype characterized by high neuroticism, high impulsivity, and low conscientiousness. This is consistent with the Personality-Health Hypothesis, which suggests that certain biological traits may condition an individual's susceptibility to the obesogenic environment. High impulsivity is associated with challenges in top-down inhibitory control, making the immediate reward of hyper-palatable food more salient than long-term health goals (28). These associations suggest that reliance on sustained self-control may be a significant burden for these individuals. Therefore, weight management approaches might benefit from shifting away from purely resistance-based strategies toward methods that modify exposure to environmental triggers and strengthen impulse regulation capacities (40).

Beyond individual psychological factors, the socio-environmental context—particularly occupational and life-stage stressors—serves as a critical upstream driver of maladaptive eating in this demographic. While psychological traits may provide internal vulnerability, external stressors arising from occupational and social environments are identified as equally salient correlates of dysregulated eating. Foremost among these, our synthesis positions the workplace as an active, structural context associated with behavioral dysregulation. The robust association between high quantitative workload, time urgency, and “utilitarian eating” (e.g., rapid consumption and substitution eating) provides evidence consistent with the Job Demands-Resources Model (41). A hypothesized mechanism for this relationship is cognitive resource depletion: high occupational demands may impose a heavy cognitive load, competing for the same neurological resources required for dietary self-regulation. When employees face relentless deadlines (11), rapid eating might represent a behavioral adjustment to maintain productivity at the expense of physiological integrity. Furthermore, substitution eating suggests a pathway where food is utilized to modulate work-related frustration (42). These findings suggest that the efficacy of workplace wellness programs focusing solely on individual behavioral change may be constrained by structural drivers (43); thus, future efforts should focus on fostering environments that protect structured meal breaks to allow for the neurobiological restoration of satiety signaling. In addition to the occupational environment, interpersonal dynamics emerged not merely as social context, but also as significant predictors of binge-eating severity, potentially linked to mechanisms of “Social Threat” (44). Unlike social support which buffers stress, peer competition and interpersonal friction trigger a specific Defeat and Entrapment response, which is evolutionarily linked to depressive states and compensatory consumption (45). Drawing on the Interpersonal Model of Binge Eating (30) and the Social Comparison Theory (46), our findings suggest that young and middle-aged adults navigating the peak pressures of career advancement are uniquely vulnerable to Status Anxiety (31). These associations imply that eating pathology in this demographic might often function as a response to social pressure. Consequently, interventions focusing on Interpersonal Psychotherapy skills (47), such as conflict resolution and navigating competitive environments, may address the hypothesized root causes of caloric surplus more effectively than dietary restriction alone.

Alongside emotional and environmental triggers, maladaptive behavioral and cognitive patterns may constitute self-perpetuating mechanisms that sustain obesity, potentially creating a cycle where physiological disruption reinforces psychological distress. These psychological and socio-environmental stressors ultimately manifest in specific, observable behavioral and lifestyle patterns. A consistent finding was the association between rapid eating, delayed meal timing, and higher BMI. This includes the link between late-night eating (after 20:00) and increased BMI, potentially mediated by disinhibition and stress (34). Furthermore, the link between rapid eating and eating past satiety (33) uggests a hypothesized “Neuro-Gut Disconnect,” where rapid consumption creates a temporal mismatch, ingesting calories faster than anorexigenic hormones can signal fullness to the brain (48). This perspective identifies eating hygiene as a tangible target for weight management. Rather than focusing solely on complex macronutrient education, simple behavioral nudges, such as extending meal duration and front-loading caloric intake, may act more directly on physiological satiety regulation (49). Finally, driving this behavioral disruption is the cognitive cycle of weight perception and dieting history. Our review reinforces the observed association where a history of rigid weight control is a strong predictor of current binge-eating severity. This aligns with Restraint Theory (50), which posits that cognitive restriction may paradoxically heighten attention to food cues, triggering a potential “What-the-Hell Effect” (counter-regulatory eating) upon minor dietary transgressions (51). Moreover, hyper-awareness of overweight status in a society permeated by weight stigma might trigger internalized anxiety rather than positive behavior change (32, 52). Consequently, clinicians might consider promoting health-enhancing behaviors without over-emphasizing weight loss as the sole metric of success. By decoupling health goals from the scale, professionals may help patients break the psychological cycle of restriction-deprivation-binge, fostering sustainable self-care rather than episodic punishment.

This review has several limitations. First, all included studies utilized a cross-sectional design, which precludes the establishment of causal relationships; the identified factors should be interpreted as correlates. Second, the reliance on self-reported questionnaires for assessing psychological states and eating patterns may introduce recall and social desirability biases. Third, the exclusion of qualitative studies may have limited our ability to fully capture the subjective lived experiences of participants. Fourth, although the systematic search was updated through November 2025, a limited number of newly published studies met our specific eligibility criteria, particularly regarding the use of validated eating behavior instruments, which may affect the representation of the most recent behavioral trends. Fifth, the significant heterogeneity of measurement tools, outcome definitions, and population characteristics across studies prevented a statistical meta-analysis and limited the direct comparability of effect sizes. Finally, although a comprehensive search was conducted, the potential for publication bias remains, as studies with non-significant findings may be underrepresented in the literature.

## 5 Conclusion

This systematic review synthesizes the multifaceted determinants associated with eating behavior among young and middle-aged adults with overweight and obesity. Our findings suggest limitations of the traditional clinical focus on caloric restriction alone, highlighting instead that dysregulated eating behaviors are shaped by a complex interplay of psycho-emotional vulnerabilities, socio-environmental stressors, and maladaptive cognitive patterns. It is important to note that these conclusions are derived from heterogeneous, predominantly cross-sectional evidence, which precludes the establishment of causal relationships. Nevertheless, the identified associative pathways underscore the potential value of moving beyond purely resistance-based dietary rules. Future research should prioritize these robustly identified psychosocial correlates to provide the high-quality data necessary for personalized interventions. Ultimately, clinical and public health strategies must adopt a broader biopsychosocial perspective, incorporating skill-based emotion regulation, mitigating structural workplace stressors, and promoting sustainable eating hygiene, to effectively support weight management and overall wellbeing in this population.

## Data Availability

The original contributions presented in the study are included in the article/Supplementary material, further inquiries can be directed to the corresponding authors.
