# Supplementary material for: Factors associated with eating behaviors of young and middle-aged adults with overweight and obesity: a systematic review
Source: Front Public Health. 2026 May 5;14:1785677. doi: 10.3389/fpubh.2026.1785677 (PMC13183636; doi:10.3389/fpubh.2026.1785677)
Supplement: Supplementary file 1 [file Table_1.docx]

**Supplementary Appendix**

**Factors influencing eating behavior of young and middle-aged adults with overweight and obesity : a systematic review**

**Yan He, YanJie Liu, WanYa Pan, WenHao Tian, Yuan Zhao, YunYu Guo, XiuQin Feng**

**Appendix 1: Search strategy and search results of different databases** (The result of Chinese databases was using Chinese language stragy)

1. PubMed:2429

#1 “Young Adult”[Mesh] or “Middle Aged”[Mesh] or youth[Title/Abstract] or young people[Title/Abstract] or Young Adults[Title/Abstract]

#2 “Obesity”[Mesh] or obese[Title/Abstract] or fat[Title/Abstract] or Overweight[Title/Abstract] or Obesity[Title/Abstract] or adipositas[Title/Abstract] or adiposity[Title/Abstract] or obesitas[Title/Abstract]

#3 "Feeding Behavior"[Mesh] or Eating Behavior*[Title/Abstract] or diet behavior*[Title/Abstract] or dietary behavior*[Title/Abstract] or dietary behavior*[Title/Abstract] or food behavior*[Title/Abstract]

#4 "Risk factors"[Mesh] or Risk Score*[Title/Abstract] or Risk Factor Score*[Title/Abstract] or Social Risk Factor*[Title/Abstract] or Health Correlates[Title/Abstract] or influencing factor*[Title/Abstract] or related factor*[Title/Abstract]

#5 #1 AND #2 AND #3 AND #4

2. Web of Science:2028

#1 Young Adult or Middle Aged or youth or young people or Young Adults

#2 Obesity or obese or Hyper nutrition or fat or Overweight or Obesity or adipositas or adiposity or obesitas

#3 Feeding Behavior or Eating Behavior or diet behavior* or dietary behavior* or food behavior* or feeding behaviors

#4 Risk factors or Risk Score* or Risk Factor Score* or Social Risk Factor* or Health Correlates or influencing factor* or related factor*

#5 #1 AND #2 AND #3 AND #4

3．Embase:1792

#1 'Young Adult'/exp or 'Middle Aged'/exp or 'youth':ab,ti or 'young people':ab,ti or 'Young Adults':ab,ti

#2 'Obesity'/exp or 'obese':ab,ti or 'fat':ab,ti or 'Overweight':ab,ti or 'Obesity':ab,ti or 'adipositas':ab,ti or 'adiposity':ab,ti

#3 'Feeding Behavior'/exp or 'Eating Behavior*':ab,ti or 'diet behavior*':ab,ti or 'dietary behavior*':ab,ti or 'food behavior*':ab,ti

#4 'Risk factors'/exp or 'Risk Score*':ab,ti or 'Risk Factor Score*':ab,ti or 'Social Risk Factor*':ab,ti or 'Health Correlates':ab,ti or 'influencing factor*':ab,ti or 'related factor*':ab,ti

#5 #1 AND #2 AND #3 AND #4

4．CINAHL:234

#1 SU (‘Young Adult’ OR ‘'Middle Aged’) OR TI (‘youth’ OR ‘young people’ OR ‘Young Adults’) OR AB (‘youth’ OR ‘young people’ OR ‘Young Adults’)

#2 SU (‘Obesity’ ) OR TI (‘obese’ OR ‘Overweight’ OR ‘fat’ OR ‘Obesity’ OR ‘adipositas’ OR ‘adiposity’) OR AB (‘obese’ OR ‘Overweight’ OR ‘fat’ OR ‘Obesity’)

#3 SU (‘Feeding Behavior’ ) OR TI (‘Eating Behavior’ OR ‘diet behavior’ OR ‘dietary behavior*’ OR ‘food behavior*’ OR ‘feeding behaviors’) OR AB (‘Eating Behavior’ OR ‘diet behavior’ OR ‘dietary behavior*’ OR ‘food behavior*’)

#4 SU (‘Risk factors’ ) OR TI (‘Risk Score*’ OR ‘Risk Factor Score*’ OR ‘Social Risk Factor*’ OR ‘Health Correlates’ OR ‘influencing factor*’ OR ‘related factor*’) OR AB (‘Risk Score*’ OR ‘Risk Factor Score*’ OR ‘Social Risk Factor*’ OR ‘Health Correlates’ OR ‘influencing factor*’ OR ‘related factor*’)

#5 #1 AND #2 AND #3 AND #4

5．Cochrane:819

#1 (Young Adult):ti,ab,kw OR (Middle Aged):ti,ab,kw OR (youth):ti,ab,kw OR (young people):ti,ab,kw OR (Young Adults):ti,ab,kw

#2 (Obesity):ti,ab,kw OR (obese):ti,ab,kw OR (fat):ti,ab,kw OR (Overweight):ti,ab,kw OR (adipositas):ti,ab,kw OR (adiposity):ti,ab,kw

#3 (Feeding Behavior):ti,ab,kw OR (Eating Behavior*):ti,ab,kw OR (diet behavior):ti,ab,kw OR (dietary behavior*):ti,ab,kw OR (food behavior):ti,ab,kw

#4 (Risk factors):ti,ab,kw OR (Risk Score*):ti,ab,kw OR (Risk Factor Score*):ti,ab,kw OR (Social Risk Factor*):ti,ab,kw OR (Health Correlates):ti,ab,kw OR (influencing factor*):ti,ab,kw OR ('related factor):ti,ab,kw

#5 #1 AND #2 AND #3 AND #4

6．CNKI：5

TKA=('青年' + '中年' + '中青年') AND TKA=('肥胖症' + '超重' + '肥胖') AND TKA=('摄食行为' + '进食行为' + '摄食方式' + '饮食行为' + '膳食行为') AND TKA=('影响因素' + '影响因素分析' + '影响因素研究')

7．Wangfang data：23

主题:(“青年” OR “中年” OR “中青年”) AND 主题:(“肥胖症” OR “超重” OR “肥胖”) AND 主题:(“摄食行为” OR “进食行为” OR “摄食方式” OR “饮食行为” OR “膳食行为”) AND 主题:(“影响因素” OR “影响因素分析” OR “影响因素研究”)
